# Multi-level barriers and facilitators to implementing tobacco screening and cessation counseling in a Federally Qualified Health Center

**DOI:** 10.1186/s12913-025-13617-5

**Published:** 2025-11-10

**Authors:** Linda Salgin, Jennifer K. Felner, Amanda Velasquez, Borsika A. Rabin, David Strong, Marva Seifert, Jerel P. Calzo

**Affiliations:** 1 The Herbert Wertheim School of Public Health and Human Longevity Science, UC San Diego, 9500 Gilman Dr, La Jolla, CA 92093 USA; 2 San Ysidro Health, 1601 Precision Park Lane, San Ysidro, CA 92173 USA; 3 School of Public Health, San Diego State University, 5500 Campanile Drive, San Diego, CA 92182 USA; 4 Dissemination and Implementation Science Center, Altman Clinical and Translational Research Institute, UC San Diego, 9500 Gilman Dr, La Jolla, CA 92093 USA; 5 Division of Pulmonary, Critical Care, and Sleep Medicine, School of Medicine, UC San Diego, 9500 Gilman Dr, La Jolla, CA 92093 USA

**Keywords:** Tobacco use cessation, Smoking cessations, Low-Income population, Implementation science, Community health center, Qualitative research

## Abstract

**Background:**

Despite persistent tobacco control efforts, the prevalence of smoking, especially among low-income populations, remains high. The prevalence of tobacco use among the primarily low-income populations served by Federally Qualified Health Centers (FQHCs), is approximately 5 percentage points higher than the national average. Evidence based interventions such as clinician delivered tobacco cessation counseling are brief and effective, however, providers are often faced with various barriers that impede their ability to offer tobacco related counseling consistently. The purpose of this study was to understand multilevel and multi-perspective barriers and facilitators to implementing tobacco screening and cessation counseling at a large, multi-site FQHC.

**Methods:**

This study used a descriptive qualitative design. Semi-structured interviews were conducted among a diverse group of FQHC staff including providers, clinical staff (e.g., nurse, medical assistant), clinic site managers, and administrators. Interviews were guided by the Practical, Robust Implementation and Sustainability Model (PRISM), lasting an average of 35 min. Data analysis included descriptive statistics to summarize participant characteristics and applied thematic analysis to identify themes related to barriers and facilitators in implementing tobacco screening and cessation counseling.

**Results:**

Sixteen FQHC staff participated in the study. Participants were between the ages of 31–69 years old (M = 47.6, SD = 11.2) and had 4–45 years of medical experience (Median = 17.25). Participants represented various roles within the FQHC with 8 Providers, 5 Administrators, and 1 Registered Nurse, Care Coordinator, and Medical Assistant each. Key themes were identified across PRISM contextual domains, including provider knowledge gaps and time constraints, patients’ motivation to quit and hesitation to disclose tobacco use, as well as external referral challenges. Prioritizing tobacco cessation alongside other important health conditions, coupled with rapport building and involving dedicated support staff in tobacco cessation efforts, were perceived to be key strategies to increase consistent delivery of tobacco cessation services.

**Conclusion:**

This study sheds light on the multifaceted barriers and facilitators to implementing tobacco cessation services within a large multi-site FQHC. By addressing these key determinants, FQHCs can further enhance ongoing efforts to reduce tobacco use among low-income communities and improve patients’ overall health.

**Title registration:**

Not applicable.

**Supplementary Information:**

The online version contains supplementary material available at 10.1186/s12913-025-13617-5.

## Introduction

In the United States (US), 15–20% of all-cause deaths can be attributed to tobacco product use [1]. Despite persistent tobacco control efforts, the prevalence of smoking, especially among low-income populations, remains high [2, 3]. Research has highlighted that populations with an income of $30,000 or less annually have 19.7–24.7% prevalence of any tobacco use, nearly double the rate of the general adult population [2, 3]. Individuals experiencing poverty are also more likely to have chronic diseases such as cardiovascular disease, obesity, hypertension, and lung cancer [4, 5] which are exacerbated by tobacco product use. Thus, these communities experience a disproportionate burden of tobacco related morbidity and mortality relative to those with higher incomes [6]. 

Federally Qualified Health Centers (FQHCs) are non-profit community health centers funded by the Health Resources and Services Administration that are typically located in medically underserved communities that offer primary care and preventative health services to all patients, regardless of their ability to pay [7, 8]. There are nearly 1,500 FQHCs across the US operating over 15,000 service delivery sites and, in 2023, FQHCs served more than 32.5 million US Americans, 90% of whom were low-income [9]. Notably, a study by Flocke et al. found that the prevalence of tobacco use among FQHC patients was 29.3%, approximately 5 percentage points higher than the national average [10]. Other studies among FQHC patients have found similar results with smoking prevalence rates between 26–30% [11, 12], underscoring the potential high impact FQHCs can have on underrepresented populations when promoting tobacco cessation [13]. 

A range of evidence-based smoking cessation strategies are available, that have been proven to be effective among diverse populations, especially those disproportionately impacted by tobacco product use, such as individuals with low incomes [14]. One of the most cost-effective methods to increase the likelihood of quitting tobacco product use includes clinician delivered tobacco cessation counseling (TCC) such as the 5 A’s (Ask, Advise, Assess, Assist, Arrange) or 3 A’s (Ask, Advise, Act/Refer) [15, 16]. TCC is a multi-step process that often first starts with screening for tobacco use. If a patient is a tobacco user, clinicians can make a strong and personalized recommendation to quitting tobacco use and refer patients to behavioral counseling and/or pharmacotherapy [14, 17]. Studies have consistently shown that patients who receive TCC are more likely to quit smoking [18–20].

With at least 70% of tobacco users seeing a provider annually [14], the literature has highlighted the importance of screening for and discussing tobacco use at every clinical encounter to maximize opportunities for screening and TCC [15, 21]. However, providers are often faced with various barriers including lack of time [13, 21,22], knowledge/awareness [21, 22] internal and external resources [21], and organizational support [23] that impede their ability to offer tobacco related counseling consistently. These challenges are further exacerbated in FQHC settings as they provide complex care to complex patients under finite resources.

Importantly, as FQHCs primarily serve patients living in poverty, who are often least likely to receive TCC [21, 24] understanding the multilevel barriers and facilitators experienced in an FQHC is an important step in identifying effective and efficient strategies to support the consistent implementation of tobacco cessation services. This study aims to elucidate key barriers and facilitators of implementing tobacco screening and cessation counseling at a large, multi-site FQHC and explore opportunities to support equitable adoption and implementation of tobacco cessation services.

## Methods

### Study design

This study used a descriptive qualitative design. Recruitment and data collection via semi-structured key informant interviews took place between October and December 2024. The San Diego State University Institutional Review Board (IRB) deemed the study not subject to IRB review. The study was approved for implementation by the partnering FQHC’s Research Review Committee (RHP-031124-169).

### Setting

The study was conducted in partnership with a large, multi-site FQHC in San Diego County. The health center operates 21 primary care facilities predominantly located in underserved and low-income communities with high rates of chronic disease and significant health disparities across San Diego’s West (*n* = 7), Central (*n* = 8), and East (*n* = 6) regions [25]. With a little over 3,000 employees, the health center provides care to approximately 150,000 patients, 90% of which live at or below 200% of the federal poverty line [25, 26]. Of the 21 clinics, 11 are represented in this study sample, with a generally balanced distribution across regions: 3 sites from the West region, 4 from Central, and 4 from East.

### Participants & recruitment

Participants for this study included health center providers, operational leaders, and front-line staff. Eligibility criteria were: (i) being over the age of 18 years old; (ii) being an active health center employee; and (iii) having primary functions that were patient facing and/or clinical site management. Based on guidance from a recent systematic review and given our homogeneous pool of participants, we expected to have sufficient data to inform qualitative analysis and generate key themes and ideas across 9–17 interviews [27]. Therefore, we estimated that a sample size of one-to-two clinicians, operational leaders and front-line staff from each of the FQHCs three regions (*n* = 3–6 per region) for a total sample ranging between 9 and 18 interviewees would us allow to gather multiple staff perspectives across the organization to achieve our analytic goal.

Participants were recruited using purposive sampling strategies, a widely used technique in qualitative research which involves identifying and selecting information-rich participants who are particularly knowledgeable about or experienced with a topic of interest, making for the most effective use of limited resources [28]. Participants were either known to the lead author as an individual engaged in tobacco related work within the health center (*n* = 11), were recommended by clinic staff (*n* = 6), or referred by other interviewees (*n* = 3), (i.e., snowball sampling). Twenty participants were contacted by the study team via email, phone, or Microsoft Teams chat function to participate in the key informant interviews of which 16 agreed. Communication included a short description of the study, purpose of interview, explanation of the study’s alignment both to the health centers clinical goals and lead author’s (LS) interests in tobacco research, as well as logistical information such as approximate interview duration. Interviews were scheduled outside of working hours (e.g., lunch, after clinic closure) with participants who agreed to serve as key informants, and were all conducted by the lead author. One day prior to the scheduled interviews, a confirmation email and study information sheet were sent to the participants.

### Data collection

A semi-structured interview guide was developed and aimed to explore multi-level barriers and facilitators in implementing tobacco cessation practices across the clinic sites. Interview questions and probes were guided by the Practical, Robust Implementation and Sustainability Model (PRISM) aligning to each of its four contextual domains: (i) intervention (organizational and patient perspectives of an intervention); (ii) recipients (organization and patient characteristics that influence intervention implementation); (iii) implementation and sustainability infrastructure (infrastructure of a given context that influences intervention implementation and sustainability); and (iv) external environment (aspects outside of the organization that can affect intervention implementation) [29]. Prior to data collection, the interview guide was piloted with two members of the study team and one health center staff. Refinements to the study guide were made to improve the clarity and flow of the interview guide. Sample questions are shown in Table 1.

**Table 1:** Sample questions from interview guide

PRISM contextual domain	Interview question
Organizational Perspective	Can you tell me about the tobacco screening and counseling quality measures that should be met by the clinics?
Organizational Perspective	Could you tell me a little bit about the process for implementing tobacco screening and counseling?
Patient Characteristic	Could you tell me a little bit about any barriers or facilitators that providers experience when delivering tobacco cessation counseling with their patients?
Patient Characteristic	Are there any patient characteristics (e.g., age, ethnicity, primary language, housing status, number of chronic conditions) that make it easier or more challenging to deliver tobacco cessation counseling?
Implementation & Sustainability Infrastructure	How does your clinic’s process for tobacco screening and cessation counseling differ from other sites or regions?
External Environment	Where would you refer your patients to get external resources for tobacco cessation services?

Interviews were held either in-person (*n* = 1) or via Zoom (*n* = 15). For in-person interviews, the lead author (LS) still scheduled a Zoom meeting and recorded the meeting to obtain audio transcription. Interviews were recorded with participants’ consent, after being assured that all responses would remain confidential and reported results would be anonymized. During each interview, electronic notes were taken and later closely reviewed by the lead author (LS) and discussed with co-authors to assess saturation of key themes and ideas. By the 14th interview, no new themes or ideas were identified in the data, prompting the collection of 2 additional interviews to ensure comprehensive data saturation. Consequently, by the 16th interview, we determined that the data had sufficiently captured all developing themes and ideas. Interviews ranged between 22 and 53 min with an average of 35 min. Each participant (except one) received a $30 gift card for participation. This one participant was required by the FQHC to decline remuneration because the interview was scheduled during their regular working hours.

### Data analysis

The analysis team was a multi-disciplinary group of junior and senior researchers with diverse ethnic backgrounds and varying experience in public health, health care, and implementation science including a research assistant (AV) with experience working in hospital settings and led by the first author (LS). The team used an applied thematic analysis approach which involved memoing and coding to identify patterns and generate themes grounded in the data [30]. 

Interview transcriptions were obtained from Zoom’s cloud recording function. Each transcript was reviewed against the audio recording by LS and AV for accuracy. Transcripts were cleaned according to a study developed protocol which included redacting any identifiable information. All data were managed and analyzed using Nvivo 14.23.4 qualitative analysis software.

First, each transcript was memo’d, a form of annotation that captures initial impressions of the data and serves as a first step in understanding the breadth and depth of the data collected [31, 32]. The lead author (LS) and research assistant (AV) were assigned specific transcripts to memo which included listening to the audio recording while following the transcript and generating notes or summaries that highlighted potential barriers and facilitator aligned with the PRISM domains along with highlighting patterns, similarities or contrasts across interviews. Second, memos were used to identify an initial set of codes (e.g., data unavailability, lack of tobacco prioritization, staffing support, age related consequence perception) that informed an initial codebook. This preliminary codebook was further refined by adding codes relevant to PRISM contextual domains (e.g. organizational characteristics, patient level barriers, external environment). The lead author (LS) and research assistant (AV) reviewed a subset of transcripts collaboratively to discuss preliminary coding decisions, identify codes that required clarification, and ensure a shared understanding of the codebook. Upon codebook finalization, the lead author (LS) and research assistant (AV) independently coded 20% of the transcripts to establish an intercoder agreement of at least 80%. Discrepancies were discussed and resolved between the coders. Once the desired intercoder agreement was achieved, the remaining transcripts were independently coded by the lead author (LS) and research assistant (AV). Finally, after all transcripts were coded, the analysis team engaged in multiple rounds of dialogue to group codes into broader patterns and identify key themes across the data. To further enhance validity of the analysis and findings, the lead author (LS) kept a detailed audit trail of analysis procedures and decisions and engaged in regular “peer debriefing” with other team members not involved in data collection or coding, with a focus codebook development and testing, and theme generation that would be meaningful to potential users of the study findings (e.g., program planners/implementers, clinical providers) [33, 34].

### Positionality & reflexivity

The lead author for this study is an employee of the FQHC with significant experience developing, implementing and evaluating research and service-oriented programs over the last decade. As a result, she has familiarity with individual and organizational barriers and facilitators that staff experience when implementing patient care workflows in high demand settings. This insider status helped inform the study focus, which aligned with organizational priorities to improve tobacco cessation efforts, along with shaping the research question, methodology, and interpretation of results. Additionally, reflexivity was used to mitigate potential bias and power dynamics in the research process. The lead author actively engaged in reflexive dialogue with co-authors to ensure that interpretations were grounded in the data rather than influenced by personal biases or preconceived assumptions.

## Results

### Participant characteristics

Among the 16 enrolled participants, 9 were female and 7 were male. Most participants (*n* = 5) identified as Hispanic/Latino or White (*n* = 5), followed by African-American (*n* = 2). Participants were between the ages of 31–69 years old (M = 47.6, SD = 11.19) and had 4–45 years of medical experience (Median = 17.25). Participants represented various roles within the FQHC with 8 Providers, 5 Administrators, 1 Registered Nurse, Care Coordinator, and Medical Assistant each. Additional participant demographic characteristics are summarized in Table 2.

### Qualitative themes by PRISM contextual domains

Qualitative results are summarized in alignment with PRISM contextual domains of the intervention, recipient, implementation and sustainability infrastructure and external environment. We note that within the PRISM contextual domain of the intervention, we do not report on the patient perspective, as data collection did not include patients. Within each contextual domain, we describe identified themes with illustrative quotes from participants (coded for anonymity as role and #). To maintain anonymity, we only describe the participants’ role.

### Intervention (Organizational perspective)

#### Lack of knowledge and awareness of guidelines and best practices hinder consistent tobacco screening and counseling

Across all participants, there was a general lack of knowledge regarding the guidelines and best practices for tobacco cessation. Two providers referenced the United States Preventative Task Force as their source for information, however, acknowledged that they could not immediately recall the specifics of those guidelines. Similarly, one manager expressed how they know evidence-based interventions for tobacco cessation exist and that they are effective, but highlighted that when staff are not using these models consistently, their details are easily forgotten stating.

It’s hard to remember the specifics of it. So the idea of like, I should ask people about their smoking use. okay, but they’re like what I’m going to do next. If there’s like an intervention that you want me to do, if I’m not doing it every day, or if I’m not doing it really consistently, it’s really hard to remember (Administrator #8)

Despite this, most participants were aware that every adult patient should get screened for tobacco use. However, across participants, analysis suggests that knowledge around how often the screening should happen and at what age screening should start was inconsistent. For instance, one manager indicated “We normally ask [patients] at every single visit. It’s not just annually. It’s at every single visit the [Medical Assistant] will actually screen patients. (Provider #7) while another shared:

The the truth is, usually we’ll reserve the tobacco counseling or screening for their wellchild visits. Yeah, so occasionally a kid comes in for something else, and if you step step a parent out then you might introduce [tobacco] as well, but usually, for I’d say 95% of that clients will happen during their annual visits (Provider #5)

About half of the participants (*n* = 8) were able to confidently indicate that the recommendation for tobacco cessation is for every patient to be screened at every visit, however, were unsure if this was actually happening in practice.

Knowledge gaps were also described by participants regarding the health effects of vaping compared to traditional cigarette use indicating a potential added challenge of being aware of guidelines and recommendations for reducing e-cigarette use. Participants noted the lack of resources on the negative impacts of vaping, with one participant commenting, “There’s not enough like public health messaging about the negative effects of vaping” (Provider #6). A second participant further connected the relationship between vaping and tobacco cessation efforts for adolescents stating, “there’s a lot of misinformation [and] I don’t have any sort of education on vaping, or the risk of vaping, which would be great, considering I have 2 pediatricians in the [clinic]” (Administrator #3). Across the data, this gap in messaging and resource availability was described by participants as affecting providers’ self-efficacy in counseling patients to reduce vaping, especially as one provider indicated their patients often rationalized their vaping behavior, stating, “it’s like better than cigarettes” (Provider #9).

#### Provider time constraints limit focus on tobacco cessation

Almost all participants identified time as the biggest barrier to consistently implementing tobacco screening and cessation counseling. Providers shared that with a 7-minute late grace period, an office visit could be as short as 13 min, offering little time to complete a full health screening and discuss all the patients concerns, let alone also focus on tobacco cessation. One provider shared.

I mean, let’s just be real. We have 20 min visits, and like that’s just not realistic to be able to address like all of the multitude of things that we could possibly be addressing for patients all the time….and so my little counseling blurbs have become shorter, briefer, and like more concise (Provider #9)

In contrast, a nurse highlighted the greater flexibility in time for screening and counseling for nurses compared to providers (i.e., physicians). When asked about time constraints as a potential barrier to TCC, they shared:

I think we have more flexibility than providers…a lot of times, doctors, they do want to spend the time, but then they realize if they spend too much time with one patient that is difficult, or has so much on their plate. Then they get behind with the others, and [it’s a] whole domino effect. So I can’t speak for all the nurses schedule, but at least for my clinic. We have enough time to spend time with whoever patient we have. (Registered Nurse #16)

From their perspective, while providers often want to dedicate more time to individual patients, competing priorities and the risk of falling behind with other appointments may prevent them from doing so.

#### Understanding the Why

Participants emphasized the importance of addressing the “why” behind tobacco cessation efforts, especially for staff who are responsible for tobacco screening. They often described how tobacco cessation is a Medical Assistant (MA) driven function, highlighting that if the MA does the screening and uploads that information into the medical record, then the provider has an indication to then follow up with cessation counseling. One provider further explained “if they do their part, it allows us to do ours. If we don’t have an MA prompt, it’s much easier for that question to get lost in the busyness of the visit” (Provider #12). Thus, for staff responsible for screening, understanding their critical role in tobacco cessation was seen to help staff move beyond viewing the process as merely a procedural checklist, and ensure consistency in both screening and documentation. One participant explained: “when you just get told it’s part of your workflow, you’re like, ‘okay, great. I’m asking my questions’….but like understanding why that matters, especially for support staff” (Administrator #8).

At the clinic level, fostering a clinic wide commitment to tobacco cessation was seen as equally important. Participants highlighted how initiatives such as “Breast Cancer Awareness” month successfully brought awareness to the need for breast cancer screening by emphasizing both patient health outcomes and organizational priorities. A similar approach for tobacco cessation was seen as an opportunity to reinforce why tobacco screening and cessation counseling was an important initiative. One clinic site manager noted:

Even things like a tobacco cessation month or a week. You know, we do that for breast cancer awareness… when you kind of get the entire clinic support staff involved, they kind of own it more (Administrator #3).

### Recipient (Patient characteristics)

#### Age-Related variability in motivations for TCC

Participants frequently shared their belief that age shaped patients’ willingness to engage in TCC. Through the data, it was evident that for younger individuals, there appeared to be a general lack of concern about the long-term consequences of smoking and minimal interest in cessation efforts. For instance, a pediatric provider stated how tobacco cessation is “not a forefront issue for teenagers…if they are smoking, they don’t seem to be very concerned about it, and they don’t seem to be very interested in quitting” (Provider #13). Similarly, two primary care providers indicated that their older adult patients, particularly those aged 70 years old and above, also conveyed reluctance to quit smoking, primarily due to having smoked throughout their entire lives. One in particular stated.

I just think that patients who are generally older have more of like a resistance to [TCC], and there’s definitely patients who are like in like their sixties and seventies like, ‘I’ve been doing this my whole life like I’m gonna die sometime like, why, stop now, who cares?’ (Provider #9)

In contrast, framing the immediate health consequences of smoking in relation to tangible, proximal concerns were identified by participants as a key motivator for engagement in TCC among middle-aged patients. A provider stated:

Once I start telling them, you know, because you smoke like the recommendation is to start screening you for lung cancer and start sending you for a CT chest. And then I get that back. And it shows like some kind of abnormality like that’s pretty motivational for my patients to quit. (Provider #5)

Participants also emphasized their beliefs that as patients get older and reach middle age they realize they are “not going to be around forever” (Provider #12) and observing abnormalities in their health outcomes as a result of smoking reinforces these fears, serving as a key push to quit smoking.

#### Patients’ tobacco use disclosure hesitation

It was often highlighted by participants that one of the reasons tobacco cessation may not be initiated is because of a patient’s hesitation to disclose their tobacco product use during the screening process. A site manager further elaborated that when Medical Assistant’s screen for tobacco use “patients don’t always want to answer…a lot of times, even though they do smoke, will say they don’t” (Administrator #2). Another participant shared.

there’s a little bit of shame associated with any kind of smoke[ing]…and I think you know, quantifying sometimes is hard…so… I think there are issues with like the screening, because I don’t think anyone wants to admit how much they they smoke (Provider #10).

This provider’s experience further portrays how the need to quantify pack years during the screening process can result in patient’s feeling uneasy or shameful regarding their smoking history and may influence patients’ openness to discuss their smoking habits.

#### Smoking as a coping mechanism

Participants shared that for some of their patients, smoking was a coping mechanism to manage their life stressors. Participants recognized that some of their patients are experiencing homelessness, financial challenges, or navigating mental health conditions, making TCC a lower priority for them. One participant reflected on their work with a tobacco cessation program implemented at their site, expressing how mental health and substance use disorders faced by their patients made it difficult to focus on tobacco cessation efforts. She explained that for the patients they work with “[They] already have all of these other stressors. I gotta find a blanket, place to sleep, something to eat. At least that cigarette will ease some of my stress, for the moment” (Administrator #11).

#### Rapport Building

Building rapport with patients was seen as a critical component for effectively engaging them in tobacco screening and cessation counseling. A pediatric provider discussed that with adolescents, building trust was essential, sharing.

I try at every visit to learn something about the patient, and I use that as a bridge to the next visit…so I think just building longitudinal relationships with these patients, I think, is going to be the biggest facilitator (Medical Assistant #15).

Participants also noted that understanding patients’ readiness to quit and linking tobacco cessation to their health were important aspects of relationship building that help lead to more personalized conversations around tobacco cessation. Many providers referenced using motivational interviewing as techniques to move patients from one stage of readiness to the next and “not pushing their agenda” (Provider #9) if patients were not ready to make a step towards quitting their tobacco use. One provider shared that gently coming back to the conversation at future visits and reminding patients that they have tools to help were good ways to bridge tobacco cessation conversations from one visit to the next. Other providers shared examples or phrases they often used to make strong yet personalized recommendations to quit smoking. For example one provider shared a tobacco related health concern of their own family member stating:

I often will say, ‘Hey, have you thought about quitting…my dad smoked for 40 years, he quit because they saw something on an x-ray so that really scared him…Are you worried about, you know, kind of the the effects of smoking?’ So sometimes personalizing, that shows that you know I kind of get it, and it also kind of just it it legitimizes their concerns or their fears they might be having that they don’t know how to verbalize (Provider #12).

Two other providers used a more simplistic approach highlighting that reducing smoking is the “single greatest thing you could do for your health at this point” (Provider #6 & #9). In either case, consistently fostering trust and connection across visits was seen as a way to strengthen patient-provider relationships and ultimately increase engagement in tobacco cessation efforts.

### Implementation and sustainability infrastructure

#### Organizational tobacco cessation prioritization

Participants shared the sentiment that tobacco cessation must be brought “to the forefront of the mind” in order to ensure consistent screening and counseling. Consistently, participants reflected on the lack of tobacco related data available to them as one gap to how tobacco cessation is not prioritized. For example, participants often cited that their quality measure scorecards included benchmarks (e.g., screening and diagnostic thresholds) for priority conditions, such as diabetes, colorectal and breast cancer screening, immunizations etc., which can be segmented at the organizational, clinic or provider level. These scorecards are reviewed monthly and can drive Plan-Do-Study-Act cycles and other initiatives to ensure that metrics are being met. Unfortunately, one provider shared “tobacco is not actually one that’s on the quality scorecard. So it’s not one that we routinely receive data back” (Provider #6) and without being able to see data related to tobacco cessation, other conditions then get prioritized. As one manger stated.

What I do feel we need is [a] measure that we could, that I could see a percentage of how many of the patients are due for the annual screening. Or they’re active smokers. And they’re due for their follow up appointment screening, you know. So I think because we have so many other measures and screenings, [tobacco] kind of gets lost in in the weeds (Administrator #7)

A second perceived gap affecting the prioritization of tobacco cessation efforts was the absence of a designated tobacco champion—someone with expertise to support and advocate for tobacco cessation initiatives across the organization. One participant reflected that having a peer to consult with was an key aspect of bringing importance to the topic of tobacco among providers. She contrasted this with women’s health screenings, such as mammograms and pap smears, which have dedicated champions and yet still require significant effort to consistently meet recommended benchmarks. Reflecting on this challenge, she questioned how tobacco cessation could ever be elevated as a priority when no such champion exists: “Can you imagine if we don’t have a champion for tobacco screening? It’s not even in our vision…we need a champion” (Provider #14).

#### Need for tobacco cessation support staff

Almost half of the participants shared how having dedicated staff to focus on TCC could not only reduce provider burden, but also provide patients with more direct contact and support. Many participants specifically reflected on how effective staff, such as Health Educators or Care Coordinators, have been in supporting patients with chronic conditions, and that using this type of model could further support tobacco initiatives. One provider described that there is a “tender moment” (Provider #6) when a patient is open to receiving further information about tobacco cessation, yet the provider needs to step away from the exam room to wrap up the clinical visit. She explained how this is a key opportunity to do a warm handoff to a Health Educator, who can reinforce counseling, offer resources, and create a care-plan (e.g., medications received, quit date, pre and post quit date follow up call, contact information for additional resources, etc.) specifically around tobacco cessation. In a similar light, another provider explained how if tobacco cessation counseling could be done by other staff, then the provider visit could focus on treatment rather than motivational interviewing.

the only thing that me as a physician, am truly truly needed for is, if there’s any kind of nicotine prescription replacements…if [TCC] could be almost like outsourced to someone else…and then now they’re like in a position where they’re ready to quit then, and they’ve already had counseling on like what’s available options, and then they’re just [with me] to talk about medication or nicotine replacement, that’d be really helpful (Provider #10)

Participants recognized that as Health Educators and Care Coordinators primarily focus on prevention and management of chronic conditions, tobacco cessation may fall outside their core responsibilities. One staff member illustrated this limitation, explaining that their role focused on referring patients to external resources rather than providing direct tobacco cessation education:

So our training is mainly on giving the flyer, making sure they scan the QR [code], and following up with the patient, making sure that someone called them from [Kick it] California. So in regards to any education in regards to smoking, I do not provide any personal education or tips on that” (Care Coordinator #4).

These varying participant perspectives highlight the potential positive impact Health Educators and Care Coordinators could have in supporting tobacco cessation efforts and reducing provider burden, while simultaneously highlighting the current limitations of their scope of work. These insights underscore the need for health centers to consider staffing options in strengthening tobacco cessation support—whether by expanding existing staff roles or introducing dedicated personnel to ensure more consistent and comprehensive patient care.

### External environment

#### Challenges with referring patients to external tobacco cessation support services

Participants discussed three overarching challenges when referring patients to external tobacco cessation support services including (i) lack of knowledge for external tobacco cessation programs, (ii) language barriers, and (iii) insurance limitations. Participants often expressed limited knowledge of external organizations outside of the quit lines, such as Kick It California or 1-800-No-Butts, that they could refer their patients to for ongoing tobacco cessation support. While some participants expressed the ease of having print out cards for the quit lines that they could hand out to patients, one participant highlighted “we don’t have a specific place to refer to easily other than the 1-800-No-Butts” (Provider #13).

Challenges with referrals were further exacerbated by language limitations making it difficult for non-English speakers to navigate cessation support services. For instance, one program manager expressed frustration that the California quitline lacked Arabic language support, especially since the prevalence of tobacco product use in that community was high. She stated “they don’t have the treatment in Arabic. That was a huge problem because we couldn’t refer the Middle East[ern] [patients], even though we did because some of them speak broken English” (Administrator #1). Language barriers were also emphasized by a second clinic site manager, who shared that while their clinic has on-site access to translation services for a broad range of languages, for patients who speak less commonly supported languages it becomes much more difficult to ensure continuity of support once referred externally. This participant rhetorically asked “what resources are there on languages outside of my clinic? I’m sure, for our communities, English and Spanish, but [what about] Tagalog, you know, [or] Mandarin, Creole” (Administrator #3).

Lastly, providers faced challenges when referring patients externally for nicotine replacement therapy. For providers who were able to conduct TCC with their patients and support them in moving from contemplation to action in regards to tobacco cessation, patients were sometimes met with insurance barriers. As a result, providers expressed feelings of frustration and failure alluding to the time spent and difficulty in getting patients to agree to start treatment in the first place and then potentially having to start over. One provider shared:

but oftentimes we just don’t know if they’ll get covered, you know if you have a patient who’s uninsured or minimally insured. I’ve had denials from patients who are insured, and it’s very frustrating to have that conversation with a patient. You try to empower them, and then it’s like denied (Provider #12)

## Discussion

Despite persistent tobacco control efforts, the prevalence of smoking among low-income communities remains high [35, 36]. While FQHCs are required to offer tobacco screening and cessation counseling as part of their preventive care services, consistent delivery remains challenging [37]. Evidence based interventions such as clinician delivered tobacco cessation counseling are brief and effective, however, providers are often faced with various barriers that impede their ability to offer tobacco related counseling consistently. The purpose of this study was to understand barriers and facilitators to implementing tobacco screening and cessation counseling at a large, multi-site FQHC. In Table 3 we summarize barriers and facilitators participants identified across the PRISM contextual domains of the intervention (organizational perspective), recipient (patient characteristics), implementation and sustainability infrastructure, and external environment. We further map these barriers and facilitators onto the Expert Recommendations for Implementing Change (ERIC) strategies [38]. These recommendations are not mutually exclusive and should be considered within programmatic constraints. We hope that these strategies can be utilized by health care organizations as starting points to support the ongoing implementation of tobacco cessation services.

Within the organizational perspective, lack of knowledge and time constraints were primary barriers for implementing tobacco cessation efforts. We found that across all participants, there was a general lack of knowledge regarding the guidelines and best practices for tobacco cessation. While many staff recognized that all adults should be screened for tobacco use, there was also inconsistency regarding the appropriate frequency and age for screening. Similar results were found by Tsui et al. 2023, where providers and clinical staff varied in their knowledge of how often tobacco screening should occur [39]. As clinical staff are often managing numerous screenings and clinical measures, strategies such as systematic prompt or chart reminders can reduce cognitive burden while increasing rates of screening and documentation [15]. This recommendation was also alluded to under the theme of “Understanding the Why” where participants noted that without a prompt from the medical assistant, discussion around tobacco often get lost in the busyness of a medical visit. Knowledge gaps were also seen regarding the health effects of vaping compared to traditional cigarettes, affecting providers’ self-efficacy in counseling patients to reduce vaping. These findings are consistent with other studies where providers have expressed although that discussions around e-cigarette use are increasing, there is a lack of empirical evidence on their harms coupled with beliefs that e-cigarettes are safer alternatives to traditional cigarettes, leading to inconsistent recommendations in continuing or quitting use [40]. With the prevalence of e-cigarette use increasing from 3.7% to 6.5%, there is a need for public health campaigns and increased education on the health implications of e-cigarette use and pragmatic tools to support staff in effectively guiding patients towards reducing e-cigarette use [41, 42]. Overall these findings stress the importance of continued training on tobacco cessation guidelines and educational materials for tobacco cessation across product types which are recommended system-level strategies and predictors for consistent implementation of tobacco screening and cessation counseling [15, 43]. 

Consistent with prior research, participants in our study cited time constraints as the primary driver for inconsistent tobacco screening and cessation counseling [22, 44, 45]. With primary care visits as short as 13 min, providers found it challenging to discuss patients’ primary concerns and also fully engage in a conversation around tobacco cessation. This aligns with findings from other studies among providers serving low-income populations, which similarly report limited time to fully screen, educate, and refer patients to needed resources [22, 46]. Conversely, time constrictions were not seen as a barrier among nursing staff. These conflicting findings suggest that with overburdened providers, utilization of non-physician staff may be an effective strategy to mitigate time constraints. The role of nurses in tobacco cessation is increasingly recognized with research highlighting their crucial role in the success of smoking cessation programs [47]. Similarly, ancillary roles such as health educators and care coordinators have been proven to support patients with managing a variety of health conditions and can be expanded to include tobacco cessation [48, 49]. As of 2024, 20 states authorize pharmacists to prescribe nicotine replacement therapy and/or tobacco cessation medications [50]. Emerging evidence suggests that collaboration with community pharmacists is another promising avenue for tobacco cessation, with one study among FQHC patients indicating that 60% of tobacco users would be comfortable talking to a pharmacist about quitting tobacco [51, 52]. With nearly 50% of providers reporting burnout, shifting the responsibility of tobacco cessation counseling to other trained clinical staff or collaboration with on-site or local pharmacists could be a useful approach to manage time limitations of providers [53]. Future research should not only aim to assess feasibility of integrating tobacco cessation into these roles, but also consider actively involving them in the designing, implementation, and evaluation of tobacco cessation interventions to enhance contextual relevance and sustainability.

Perceived patient-level characteristics that were observed to make implementation of tobacco cessation challenging including variations in motivation to quit smoking, hesitation to disclose tobacco use, and smoking as a coping mechanism. Participants noted that adolescents and older adults were more reluctant to discuss tobacco cessation compared to their middle-aged peers. Cultural factors, shame, and stigma also were perceived to contribute to patients’ reluctance to disclose their tobacco use, making it difficult to initiate screening or counseling. Additionally, smoking was identified as a coping strategy for patients dealing with daily stressors such as food insecurity and housing instability, making tobacco cessation a lower priority. This combination of cultural factors and social needs as barriers to engaging in tobacco cessation are recognized in similar studies [39, 46]. More broadly, the literature has consistently demonstrated that minoritized and low-income populations are less likely to be screened for and counseled to quit tobacco [24, 54–56], and our results provide insights into potential underlying reasons. Building rapport with patients, offering education to dispel misconceptions about continuing tobacco use and cessation, as well as using personalized recommendations that consider their health and life circumstances can help overcome these barriers and support patients in increasing their readiness to quit [13].

Regarding the implementation and sustainability infrastructure, participants emphasized the need to prioritize tobacco cessation in the same way that chronic health conditions are prioritized. They identified the lack of tobacco-related data accessible in their quality measure scorecards as a significant barrier. Similar findings were reported by Pestka and colleagues who highlighted a need for shared goals and cooperation across the health system [57]. Specifically, their study noted that as tobacco use contributes to various health issues, reminders for screening should be as frequent, if not more so, than those for other conditions such as diabetes [57]. System level strategies such as audit and feedback where tobacco-related performance data are consistently collected, summarized, and shared with clinical staff are one recommendation that health centers can adopt to support continued for monitoring and evaluation of tobacco cessation efforts [15, 58]. Additionally, the absence of a designated tobacco champion within the organization was seen as a gap, underscoring the importance of having an expert advocate to support tobacco cessation initiatives. Tobacco Treatment Specialists (TTS) are one example of trained experts who deliver intensive tobacco treatment interventions that can serve as champions to support tobacco cessation efforts within health systems [15, 59]. Research has shown that TTS training improves knowledge and self-efficacy to deliver tobacco treatment among providers and notably; emerging evidence highlights the promise of nurses and other non-medical staff with TTS training in helping tobacco users quit [60–62]. Thus, health systems aiming to strengthen tobacco cessation efforts may benefit from integrating data sharing approaches and designating tobacco champions such as TTS. Without these support systems other priority health measures may continue to overshadow tobacco cessation efforts.

Lastly, within the external environment, participants shared various examples of challenges with referrals to external tobacco cessation support services. In line with research by Matthews et al., and Lui et al., participants shared limited knowledge of external organizations beyond quit lines they could refer patients to, language barriers among those referred to external resources, and insurance limitations for patients seeking nicotine replacement therapy [13, 22, 24]. We note that among our participants, insurance related barriers were primarily associated with prior-authorizations requirements or denials due to incomplete documentation. While no participants identified patients’ fear of losing health insurance or facing increased premiums due to tobacco use, we acknowledge that only eight states, including California, currently prohibit tobacco surcharges which may be a barrier for patients seeking tobacco treatment in other states [63]. Nonetheless, creating partnerships with local community based organizations along with a streamlined referral process to state quit lines coupled with bi-directional data sharing can help overcome some of these challenges [22].

### Strengths and limitations

This study has several strengths. First, data were collected within a large multi-site FQHC serving urban, sub-urban, and rural communities allowing for a broad understanding of tobacco cessation efforts across regions. Second, we included multiple perspectives from providers, clinic site managers, and front-line staff (i.e., Registered Nurse, Care Coordinator, Medical Assistant, etc.), which have previously been lacking in the literature. Third, the lead author is a longstanding employee of the organization whose rapport with participants may have enabled more openness in participants’ responses. Lastly, we tie findings to well-known implementation strategies that are pragmatic for FQHC settings to facilitate tobacco cessation initiatives. However, there are limitations to consider. FQHCs are unique systems, and thus these data may not be generalizable to other FQHCs. We did not include patients in interviews limiting our understanding of barriers and facilitators from the patient’s perspective. Similarly, we did not interview pharmacists, nurse practitioners, or physician assistants who may encounter distinct or overlapping barriers and facilitators to implement tobacco cessation efforts, which could have further enhanced our understanding of staff who may be more accessible for delivering cessation support. We also acknowledge that while an overall strength, the lead author’s positionality may have led to potential response bias. Additionally, as the lead author conducted all interviews and was closely involved in data interpretation, there is a potential risk for investigator bias. Nonetheless, this study sheds light on the key multi-level determinants of implementing tobacco screening and cessation counseling within the context of FQHCs.

## Conclusion

This study provides valuable insights on the multifaceted barriers and facilitators affecting delivery of tobacco cessation service within a large multi-site FQHC. Key themes identified across PRISM contextual domains, including provider knowledge gaps and time constraints, patients’ motivation to quit and hesitation to disclose tobacco use, as well as external referral challenges. Prioritizing tobacco cessation alongside other important health conditions, coupled with rapport building and involving dedicated support staff in tobacco cessation efforts, were perceived to be key strategies to increase consistent delivery of tobacco cessation services. By addressing these key determinants, FQHCs can further enhance ongoing efforts to reduce tobacco use among low-income communities and improve patients’ overall health.

**Table 2:** Participant (Recipient) demographic characteristics (*n* = 16)

Demographic characteristic	*N* (%)
**Age (mean**,** SD**,** range)**	47.6, [11.2], 31–69
**Years of Medical Experience (median**,** range)**	17.25, 4–45
**Gender**	
Female	9 (56.25%)
Male	7 (43.75%)
**Ethnicity**	
Black/African-American	2 (12.5%)
Biracial	1 (6.25%)
Hawaiian/Pacific Islander	1 (6.25%)
Hispanic/Latino	5 (31.25%)
Middle Eastern	1 (6.25%)
White	5 (31.25%)
Vietnamese	1 (6.25%)
**Highest Level of Education**	
Less than Bachelor’s Degree	3 (18.75%)
Bachelor’s Degree	2 (12.5%)
Master’s Degree	3 (18.75%)
Medical Degree (MD/DO)	8 (50%)
**San Diego Region**	
Central	5 (31.25%)
East	5 (31.25%)
West	6 (37.5%)
**Primary Role**	
Administrator (Manager, Director)	5 (31.25%)
Care Coordinator	1 (6.25%)
Medical Assistant	1 (6.25%)
Provider (MD/DO)	8 (50%)
Registered Nurse	1 (6.25%)

**Table 3:** Summary of results with accompanying expert recommendations for implementing change (ERIC) strategies

PRISM Contextual Domain	Theme	Barriers/Facilitators identified	Potential ERIC Implementation Strategy to address barrier/facilitator
Intervention (Organizational Perspective)	Lack of knowledge and awareness of guidelines and best practices hinder consistent tobacco screening and counseling	1. Limited knowledge of general tobacco cessation guidelines & best practices. 2. Limited knowledge and resources for the health effects of vaping, making it difficult counsel patients on reducing e-cigarette use.	1. Conduct Ongoing Training: Plan for and conduct tobacco cessation training at least annually through Learning Management System and/or in-person. 2. Develop Educational Materials: Develop manuals, toolkits, or other supporting materials around tobacco cessation guidelines and best practices 3. Distribute Educational Materials: Share educational materials in multiple formats (e.g., in-person, e-mail)
	Provider time constraints limit focus on tobacco cessation	1. Limited time in office visit to complete full health screening, discuss patient and provider concerns, and address tobacco use.	1. Revise Professional Roles: Revise and/or shift roles among existing Health Educators/Care Coordinators or Nurses to discuss tobacco cessation
	Understanding the why	1. Emphasizing the “why” behind tobacco cessation efforts is key to moving away from seeing the process as a checklist item. 2. Important to foster clinic wide commitment to tobacco cessation.	1. Remind Clinicians: Develop reminder systems to help staff to engage in tobacco cessation efforts 2. Conduct Educational Meetings: Hold meetings targeted towards providers, administrators, and other organizational staff to discuss importance of tobacco cessation efforts
Intervention (Patient Characteristics)	Age-related variability in motivations for tobacco cessation counseling	1. Adolescents and older adults were perceived to be more reluctant to discuss quitting tobacco relative to their middle-aged peers.	1. Tailor Strategies: Tailor tobacco cessation efforts to address patient level barriers 2. Prepare Patients to be Active Participants: Identify opportunities to actively engage patients in understanding tobacco cessation efforts 3. Capture and Share Local Knowledge: Share best practices on how to overcome patient level barriers
	Patients’ tobacco use disclosure hesitation	1. Cultural factors, shame, and stigma were perceived to contribute to patients’ reluctance to disclose their tobacco use, making it difficult to initiate screening or counseling.	1. Tailor Strategies: Tailor tobacco cessation efforts to address patient level barriers. 2. Prepare Patients to be Active Participants: Identify opportunities to actively engage patients in understanding tobacco cessation efforts. 3. Capture and Share Local Knowledge: Share best practices on how to overcome patient level barriers.
	Smoking as a coping mechanism:	1. Smoking was identified as a coping strategy for patients dealing with daily stressors making tobacco cessation a lower priority.	1. Tailor Strategies: Tailor tobacco cessation efforts to address patient level barriers. 2. Prepare Patients to be Active Participants: Identify opportunities to actively engage patients in understanding tobacco cessation efforts. 3. Capture and Share Local Knowledge: Share best practices on how to overcome patient level barriers.
	Rapport building	1. Building longitudinal relationships with patients is necessary to engage them in tobacco cessation.	1. Tailor Strategies: Tailor tobacco cessation efforts to address patient level barriers. 2. Prepare Patients to be Active Participants: Identify opportunities to actively engage patients in understanding tobacco cessation efforts.
Implementation and Sustainability Infrastructure	Organizational tobacco cessation prioritization	1. Lack of tobacco-related data points within quality scorecards along with absence of tobacco champion were seen hinder tobacco cessation efforts.	1. Audit & Feedback: Collect and summarize tobacco-related performance data and provide it to provide it to physicians and clinical administrators to monitor and evaluate. 2. Facilitate Relay of Data: Provide close-to-real-time data on tobacco screening, counseling, and referral outcomes. 3. Identify and Prepare Champions: Identify an individual who can dedicate themselves to advocating for tobacco cessation and providing expertise.
	Need for tobacco cessation support staff	1. Health Educators/Care Coordinators are supportive roles that can facilitate tobacco cessation efforts while reducing provider burden.	1. Revise Professional Roles: Revise and/or shift roles among existing Health Educators/Care Coordinators to discuss tobacco cessation.
External Environment	Challenges with referring patients to external tobacco cessation support services	1. Limited knowledge of external organizations to refer patients to for tobacco cessation counseling. 2. Language barriers made using quit lines difficult for less commonly spoken languages 3. Referrals for nicotine replacement therapy being hindered by insurance.	1. Develop & Distribute Educational Materials: Develop and share local resources for tobacco cessation within community in multiple formats (e.g., in-person, e-mail). 2. Develop Resource Sharing Agreements: Develop partnerships with local organizations (including quit line) to support ongoing tobacco cessation efforts.

## Supplementary Information

Below is the link to the electronic supplementary material.

Supplementary Material 1

## Data Availability

The datasets generated and/or analyzed during the current study are not publicly available due organizational policies, but may be available from the corresponding author on reasonable request.

## Abbreviations

FQHC: Federally Qualified Health Center
IRB: Institutional Review Board
PRISM: Practical, Robust Implementation and Sustainability Model
TCC: Tobacco Cessation Counseling
