# Supplementary material for: Multi-level barriers and facilitators to implementing tobacco screening and cessation counseling in a Federally Qualified Health Center
Source: BMC Health Serv Res. 2025 Nov 10;25:1457. doi: 10.1186/s12913-025-13617-5 (PMC12604286; doi:10.1186/s12913-025-13617-5)
Supplement: Supplementary file 1 — Supplementary Material 1 [file 12913_2025_13617_MOESM1_ESM.docx]

**Qualitative Interview Guide**

**Interview Questions – PRISM Domain: Organization Perspective Block**

1. Could you tell me a little bit about yourself and your role with [health center name]?

For the next question, I’d like to ask you about the guidelines at the national level and how they impact your work.

1. Can you tell me about any tobacco cessation guidelines you’re familiar with at the national level that might have impact on how we provide tobacco cessation services locally?
   1. *Probe 1*: What makes these guidelines easy or difficult to use?
   2. *Probe 2*: What are some of the challenges you experience with staying up to date on changes to these guidelines and/or refreshing yourself on these guidelines?
2. Can you tell me about the tobacco screening and counseling quality measures that should be met by the clinics?
   1. *Probe 1*: Does your clinic experience any challenges in meeting these metrics?
      1. If yes, Could you tell me about some of those challenges
      2. If no, Could you tell me about some of the factors that allow you all to meet these metrics?
3. I’m trying to understand how different sites implement tobacco cessation services across [health center name]. Could you tell me a little bit about the process for implementing tobacco screening and counseling?
   1. *Probe 1*: What tobacco or nicotine products are patients typically screened for?
   2. *Probe 2*: Which positions at your clinic typically play a role in screening for and implement tobacco cessation counseling?
   3. *Probe 3*: What are some of the challenges that come up for these roles when screening for and implement tobacco cessation counseling with patients.
   4. *Probe 4:* What training or resources do you think are needed to ensure that staff of different levels (e.g, MA, Nurse, MD) can consistently screen for and implement tobacco cessation counseling?
4. Could you tell me a little bit about the process for documenting tobacco screening and cessation counseling in the patient's EHR record?
   1. *Probe 1*: What are some of the challenges that come up when documenting tobacco screening or cessation counseling.
   2. *Probe 2:* What training or resources are needed to ensure that screening and counseling is documented appropriately in the EHR?

**Interview Questions – PRISM Domain: Patient Characteristic Block**

For these next questions, I’d like to ask you specifically about tobacco cessation counseling. First, I will ask about your experience with delivering cessation counseling to patients and what makes it easy or difficult to do so. Then, I will ask about how delivery of tobacco cessation counseling may differ for patients with different sociodemographic and health factors. So to start,

1. Could you tell me a little bit about any barriers or facilitators that providers experience when delivering tobacco cessation counseling with their patients? Essentially what makes it easy or difficult to implement tobacco cessation counseling with your patients.
2. Are there any patient characteristics (e.g., age, ethnicity, primary language, housing status, number of chronic conditions) that make it easier or more challenging to delivering tobacco cessation counseling
   1. *Probe 1:* How does the implementation of tobacco cessation screening and/or counseling differ between adolescents and adults?
   2. *Probe 2*: What are some of the challenges that you (or your staff) experience when implementing tobacco screening or cessation counseling with patient's whose primary language is not English?
   3. *Probe 3*: What challenges do you see when trying to address tobacco and nicotine use among patients experiencing homelessness?
   4. *Probe 4*: When working with these patients with multiple comorbidities, how do you (or your clinicians) balance prioritizing their medical needs while also screening for tobacco and offering cessation counseling?
      1. Probe 4a: How does the prioritization of tobacco cessation counseling change based on the type of medical condition patients are experiencing - for example, a patient who uses substances vs. patients who are diabetic?

**Interview Questions – PRISM Domain: Implementation & Sustainability Infrastructure Block**

1. Thinking about the region that you work in, how does your clinic’s process for tobacco screening and cessation counseling differ from other sites or regions?
   1. *Probe 1:* What are some of the things that are working well in your clinic that might be useful in other clinics/regions as it relates to tobacco screening and cessation counseling?
   2. *Probe 2*: From a broader organizational perspective, what resources are needed to successfully implement tobacco screening and cessation services consistently across all primary care sites?

**Interview Questions – PRISM Domain: External Environment Block**

1. Where would you refer your patients to get external resources for tobacco cessation services?
